# NRF2-regulated cell cycle arrest at early stage of oxidative stress response mechanism

**DOI:** 10.1371/journal.pone.0207949

**Published:** 2018-11-28

**Authors:** Margita Márton, Nikolett Tihanyi, Pál Gyulavári, Gábor Bánhegyi, Orsolya Kapuy

**Affiliations:** 1 Department of Medical Chemistry, Molecular Biology and Pathobiochemistry, Semmelweis University, Budapest, Hungary; 2 MTA-SE Pathobiochemistry Research Group, Budapest, Hungary; University of South Alabama Mitchell Cancer Institute, UNITED STATES

## Abstract

Oxidative stress results in activation of several signal transduction pathways controlled by the PERK-substrate NRF2 (nuclear factor erythroid 2-related factor 2); meanwhile the ongoing cell division cycle has to be blocked. It has been recently shown that Cyclin D1 got immediately down-regulated via PERK pathway in response to oxidative stress leading to cell cycle arrest. However, the effect of NRF2 on cell cycle regulation has not been explored yet. We aimed to reveal a crosstalk between PERK-substrate NRF2 and the key elements of cell cycle regulatory network upon oxidative stress using molecular biological techniques- Although Cyclin D1 level remained constant, its activity was blocked by various stoichiometric inhibitors (such as p15, p21 and p27) even at low level of oxidative stress. The activity of these CDK inhibitors completely disappeared, when the addition of oxidative agent was combined with silencing of either PERK or NRF2.This further confirms the important role of NRF2 in blocking Cyclin D1 with stoichiometric inhibitors at early stage of oxidative stress.

## Introduction

From internal metabolism and external toxicant exposure several harmful reactive oxidants (such as reactive oxygen (ROS) and nitrogen (RNS) species) might be formed generating oxidative stress in the cell. A significant decrease in the antioxidant (e.g. glutathione) controlled cellular defence mechanism can also lead to oxidative stress [1]. The highly reactive ROS and RNS molecules can be generated even at physiological conditions formed in a well-controlled manner, and they are also used by the immune system as a way to remove pathogens [2]. In humans, however hyper-production of these reactive oxidants might have fatal consequences by inducing serious diseases [3, 4]. Oxidative stress-generated effects are involved in neurodegenerative diseases (e.g. Parkinson’s disease), sickle-cell disease, toxicity of xenobiotics, heart failure and cancer development [3, 4]. Therefore oxidative stress response mechanisms have to be highly controlled [2].

The nuclear factor erythroid 2-related factor 2 (NRF2) has a key role to enable adaptation to oxidative stress [5, 6] by transcriptionally controlling more than 2000, mainly cytoprotective genes [7]. The induction of NRF2 genes requires a common NRF2-binding motif on the DNA, known as antioxidant response element (ARE) or electrophile response element (EpRE) [8, 9]. NRF2 also directly reduces the level of both ROS and RNS by promoting the expression of their suppressors (*i*.*e*. catalase or nitric oxide synthase) [6]. Furthermore, NRF2 induces the expression of other protective genes encoding phase I and II detoxification enzymes, transport proteins, proteasome subunits, the regulators of various cell death mechanisms, antioxidant proteins, growth factors, chaperones and other transcription factors [1, 10, 11].

It is well-known that mimicking oxidative stress by H_2_O_2_ or TBHP (tert-butyl-hydroperoxide) treatment results in activation of several signal transduction pathways of that stress response mechanism; meanwhile the ongoing cell division cycle has to be blocked [12, 13]. At physiological conditions the cell creates a precise duplication of its DNA content in S phase; meanwhile segregation of sister chromatids occurs during mitosis (M phase) [14, 15]. S and M phases are separated by gap phases, so called G1 (between M and S) and G2 (between M and S) phases, respectively [14]. The maintenance of appropriate oscillation of these cell cycle phases is controlled by a complex protein regulatory network [16]. The key protein molecules of the cell cycle machinery are the cyclin dependent kinases (Cdks) [14]. The level of Cdks remains relatively constant throughout the cell cycle, therefore their activity is controlled by various posttranslational modifications (i.e. cyclin binding, cyclin activated kinase phosphorylation, regulatory phosphorylation and/or binding of Cdk inhibitor) [14, 17].

It is well-known that D-type cyclin has an essential role in controlling the start event of cell cycle by binding Cdk4 or Cdk6 at physiological conditions [18, 19]. Besides that, many scientific data have revealed that Cyclin D1 also plays an important role in G2 phase [20, 21]. However, Cyclin D1 seems to be sensitive to a variety of extracellular stimuli. It has shown that activity of Cyclin D1 level is also modified by oxidative stimuli [22, 23]. It has been recently clarified that high level of ROS induced by H_2_O_2_ treatment has a significant effect on the regulation of D-type cyclin in human cells [12]. Cyclin D1 depletion is quickly detected after addition of oxidative stressor, meanwhile the cells become arrested in G2 phase. It is also confirmed that Cyclin D1 is linked to the G2/M transition via the Chk1-Cdc2 DNA damage checkpoint pathway in ROS-induced cell cycle block [12]. Interestingly, the down-regulation of Cyclin D1 is highly controlled by one arm of the unfolded protein response mechanism (UPR), called PERK pathway [12]. Namely, eiF2α, the direct target of PERK, seems to be responsible for Cyclin D1 depletion in response to oxidative stress [12].

As it has been already shown, NRF2 is also a PERK substrate. The nuclear translocation of NRF2 occurs in a PERK dependent manner with respect to endoplasmic reticulum (ER) stress [24, 25]. In the absence of PERK the induction of NRF2 substrates (such as NRF2 target NAD(P)H:quinone oxidoreductase 1 or NQO1 for short) is not detected [24]. Nrf2-/- cells are sensitive to variety of ER stress inducing agents, while NRF2 overexpression enhances cell survival suggesting that NRF2 signalling pathway is required for survival during ER stress-induced UPR activation [25].

Since NRF2 is the key element of oxidative stress response mechanism and also a PERK substrate, a question immediately arises, whether NRF2 has a role in controlling cell cycle arrest via Cyclin D1 down-regulation upon oxidative stress. Here in this report we describe a novel role of NRF2 in controlling cell cycle machinery by inhibiting Cyclin D1 throughout its stoichiometric inhibitors at the initial stage of mild oxidative stress.

## Materials and methods

### Materials

Tert-Butyl hydroperoxide solution (Sigma-Aldrich, Luperox TBH70X, 458139) was purchased. All other chemicals were of reagent grade.

### Cell culture and maintenance

As a model system, human embryonic kidney (HEK293T, ATCC, CRL-3216) cell lines were used, and maintained as described in detail previously [26]

### Cell counting

After each treatment, the relative amount of viable cells was calculated by LUNA Automated Cell Counter (Logos Biosystems). The cell counting was preceded by staining them with trypan blue (0.4% stain, Logos Biosystems).

### Sodium dodecyl sulfate polyacrylamide gel electrophoresis (SDS-PAGE) and Western blot analysis

Cells were harvested and lysed and protein content of cell lysates was measured as described previously [26] Whole cell lysates were separated by SDS-PAGE (in 10% or 15% SDS-polyacrilamide gel). SDS-PAGE and Western Blot analysis was done according to our previous study [26]The following antibodies were applied: anti-NQO1 (Cell Signaling, A180), anti-CyclinD1 (Santa Cruz, A-12), anti-p21 (Santa Cruz, C-19), anti-p27 (Santa Cruz, C-19), anti-p15-INK4b (proteintech, 12877-1-AP), anti-p16-INK4A (proteintech, 10883-1-AP), anti-PERK (Cell Signaling, 3192S), anti-P-eIF2α (Cell Signaling, 9722S9), anti-P-eIF2α (Ser51) (Cell Signaling, 9721L), and anti-GAPDH (Santa Cruz, 6C5) and HRP conjugated secondary antibodies (SantaCruz, sc-2020 and Cell Signaling, 7074S, 7076S).

### Statistics

For densitometry analysis, Western blot data were acquired using ImageJ software, while the statistical analysis was performed as described previously [26].

### RNA interference

RNA interference experiments were performed as it was previously described [27].The siPERK and siNRF2 oligonucleotides were purchased from Eurofins Genomics (the oligonucleotide sequence: siPERK: 5’-GUGACGAAAUGGAACAAGA(dTdT)-3’; siNRF2: 5’-GGUUGAGACUACCAUGGUU (dTdT)-3’).

### Reverse transcription-polymerase chain reaction (RT-PCR)

Total RNA content of cells was extracted using TRIzol RNA isolation reagent (Invitrogen) [28]. Retrotranscription and real-time PCR was performed as we previously described [27] PCR reaction and real-time detection was performed using PowerUp SYBR Green Master Mix (Thermo Fisher Scientific, A25742) and QuantStudio 12K Flex System (Thermo Fisher Scientific). The real-time PCR thermocycles were the followings: 95°C 10 min (1x), 95°C 15 s, 60°C 1 min (40x), 95°C 15 sec, 60°C 1 min, 97°C 15 s (1x). The primers were as follows: for PERK: (forward) 5’-AAAGCAGTGGGATTTGGATG-3’ and (reverse) 5’-TCTTGGTCCCACTGGAAGAG-3’, for NRF2: (forward) 5’-TCCAGTCAGAAACCAGTGGAT-3’ and (reverse) 5’-GAATGTCTGCGCCAAAAGCTG -3’, for GAPDH: (forward) 5’-TGCACCACCAACTGCTTAGC-3’ and 5’-GGCATGGACTGTGGTCATGAG-3’.

### Cell cycle analysis

HEK293T cells were incubated with 100 μM TBHP for 3 hours and with 30 μM TBHP for 6 hours (at the density of 6*10^5^cells/ml). For cell cycle analysis, all floating and adhering cells were trypsinized and then washed two times with cold PBS. Cells were fixed with 70% ethanol at -24°C overnight. Fixed cells were washed twice with PBS and then were treated with RNase A (Thermo Fisher Scientific, EN0531) in the concentration of 10 μg/ml for 30 minutes at 37°C. After incubation, cells were washed again with PBS and were re-suspended with 200 μl PBS containing 40 μg/ml propidium iodide (Thermo Fisher Scientific, P3566) and analysed by flow cytometry. Cell cycle analysis was performed on FACSCalibur (BD Bioscience) and the percentage of cells in the different phases of cell cycle was calculated by FlowJo software.

## Results

### Cyclin D1 level is not decreasing at early stage of low level of oxidative stress

**Fig 1:**
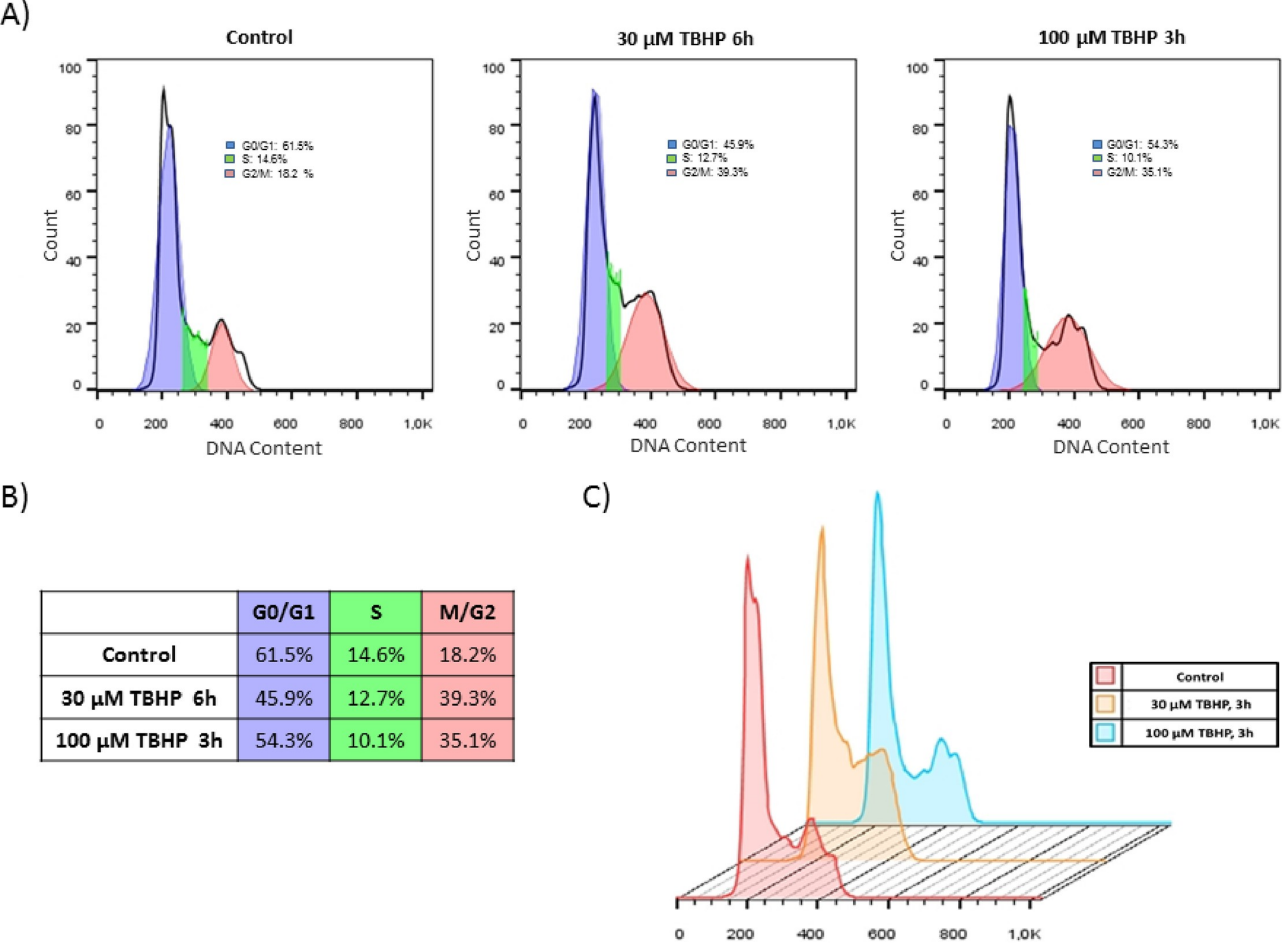
Cell cycle block gets induced even at early stage of low level of oxidative stress. Flow cytometry cell cycle analysis of TBHP-treated HEK293T cells. **(A)** Cells were treated with 30 μM TBHP for 6 hours and 100 μM TBHP for 3 hours. After fixation, cell cycle analysis was performed with propidium iodide staining. Curve fitting and calculating the different phases of cell cycle were measured by FlowJo Software. **(B)** Summary table of the cell cycle distribution in different samples. **(C)** Overlay histogram of the cell cycle analysis was also made by FlowJo Software.

In order to test the effect of NRF2 in cell cycle regulation first we set up a protocol to induce oxidative stress in human embryonic kidney cells (HEK293T) by using tert-butyl hydroperoxide (TBHP). TBHP is widely used to generate oxidative environment by reducing glutathione level, peroxidising cellular lipids and altering cellular Ca^2+^ homeostasis [29, 30]. Our goal was to introduce various levels of oxidative stress. By our definition mild oxidative stress does not induce cell death mechanism; while excessive level of oxidative stress turns on the self-killing mechanism. To verify whether TBHP treatment modifies cell cycle machinery, FACS analysis was carried out by using propidium iodide staining (Fig 1). HEK293T cells were cultured in the presence of 30 μM TBHP for 6 hours and 100 μM TBHP for 3 hours. A remarkable tendency to cell cycle arrest was already observed after 3 hours assuming a relatively quick cellular oxidative stress-response mechanism. The propidium iodide staining indicated that cell cycle machinery already answered to a minimal oxidative treatment, but full cell cycle block cannot be observed in this case. Therefore we can distinguish an early stage of treatment when the cell response mechanism is just turned on in oxidative stress.

**Fig 2:**
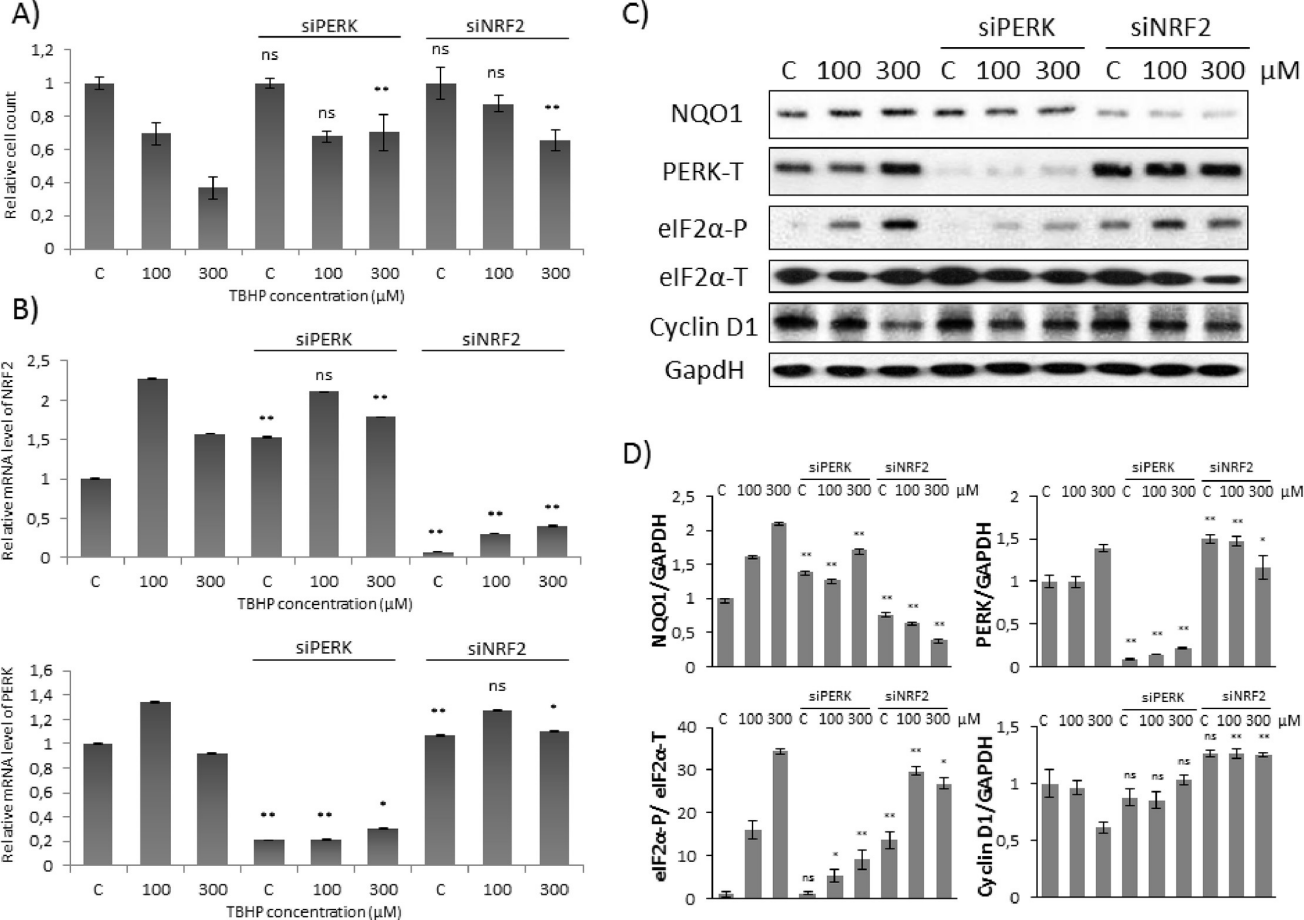
Cyclin D1 degradation is not observed at early stage of low level of oxidative stress. Concentration dependency of oxidative stress treatment was followed with/without a background of PERK or NRF2 silencing. HEK293T cells were cultured with 100 or 300 μM TBHP for 1.5 hours, while *NRF2* or *PERK* gene expression was depleted by *NRF2* or *PERK* siRNA. (**A)** The relative amount of viable HEK293T cells. **(B)** The efficiency of NRF2 (upper panel) and PERK (lower panel) silencing was checked on mRNA level. The mRNA level was followed by real-time PCR. GAPDH was used as a housekeeping gene. The intensity of NRF2 is normalised for GAPDH. **(C)** During oxidative stress the markers of NRF2 (NQO1), PERK (PERK-T, eiF2α -P) and Cyclin D1 were followed by immunoblotting. GAPDH was used as a loading control. **(D)** Densitometry data represent the intensity of NQO1, PERK-T and Cyclin D1 normalised for GAPDH and eiF2α-P normalized for total level of eiF2α. For each of the experiments, three independent measurements were carried out. Error bars represent standard deviation; asterisks indicate statistically significant difference from the control: * p < 0.05; ** p < 0.01.

Although significant cell cycle arrest was not detected on population level upon short oxidative stress, we intended to check whether cell cycle machinery has already started to induce its response mechanism on molecular level. Since Pyo et al. has shown that Cyclin D1 protein level got rapidly diminished during H_2_O_2_ treatment, Cyclin D1 was also detected by immunoblotting (Fig 2C and 2D). Excessive level of oxidative agent (300 μM TBHP for 1.5 hour) significantly decreased the amount of Cyclin D1 in the cells further confirming that the absence of Cyclin D1 might have a key role to block cell cycle at G2 phase during oxidative stress. However this drastic drop of Cyclin D1 level was not detected when cells were treated with lower concentration of TBHP (Fig 2C and 2D).

These results imply that diminish of Cyclin D1 is not required at early stage of cell cycle response upon mild oxidative stress. Rather the control network requires some other mechanism to block cell cycle progressing.

### NRF2 is essential for cell cycle block at early stage of excessive oxidative stress

Next we explored the effect of both PERK and NRF2 during TBHP treatment. Although the relative amount of viable cells was not affected at mild oxidative stress, 300 μM TBPH could induce a significant increase in cell viability during either NRF2 or PERK silencing (Fig 2A). Oxidative stress generated by addition of 100 μM TBHP for 1.5 hours was enough to enhance the level of both NRF2 and PERK mRNA (Fig 2B). However 300 μM TBHP for 1.5 hours did not increase neither NRF2 nor PERK mRNA levels (Fig 2B). Interestingly, a well-known NRF2 substrate, called NQO1 and also a PERK target, eiF2α-P, resulted in a drastic elevation on protein level upon either 100 or 300 μM TBHP treatments supposing that both NRF2 and PERK got activated both at mild and excessive levels of short oxidative stress (Fig 2C and 2D).

Western blot experiments were also performed when TBHP treatment was preceded by silencing of PERK with siRNA (Fig 2B). In this case Cyclin D1 level did not change after 1.5 hours long treatment with 100 μM TBHP, however the level of Cyclin D1 also remained high after addition of 300 μM TBHP. Our data confirm that PERK is essential to promote the induction of Cyclin D1-dependent cell cycle arrest at high level of oxidative stress, although we could not verify whether PERK has a role or not in controlling this process at short treatment with 100 μM TBHP. Interestingly, similar results were observed when NRF2 was silenced by using siRNA, namely Cyclin D1 level remained high upon excessive level of oxidative stress (Fig 2C and 2D). Although eiF2α-P was significantly increased in these cells, in the absence of NRF2 Cyclin D1 level did not decrease at all. Our data suppose that eiF2α-P alone is not sufficient to promote Cyclin D1 degradation rather NRF2 has some effect on it upon high level of oxidative stress. However Cyclin D1 expression seems to be not affected when NRF2 was silenced by using siRNA at low level of TBHP treatment.

Our data suggest that beside PERK-induced eiF2α-P, another PERK substrate, NRF2 also has an essential role in down-regulating Cyclin D1 and therefore it promotes the initiation of cell cycle arrest at excessive level of oxidative stress.

### Silencing of PERK results in depletion of Cyclin D1 inhibitors during low level of oxidative stress

Our results confirm that both PERK and its target NRF2 are essential at treatment with excessive level of oxidative agent to down-regulate Cyclin D1. However our data also indicate that Cyclin D1 level is not affected at early stage of mild oxidative stress. In our lab it got emerged that this key cyclin of cell cycle machinery is regulated with a much quicker process (e.g. by posttranslational modification), as well. It is well-known that Cyclin D1 is controlled by various inhibitors at the different phases of cell cycle progress. While stoichiometric inhibitors p15 and p16 have essential roles in G1 phase; both p21 and p27 inhibit Cyclin D1 in G2 phase [31–33]. Therefore we assume that Cyclin D1 activity is controlled by some of these inhibitors at early stage of oxidative stress.

**Fig 3:**
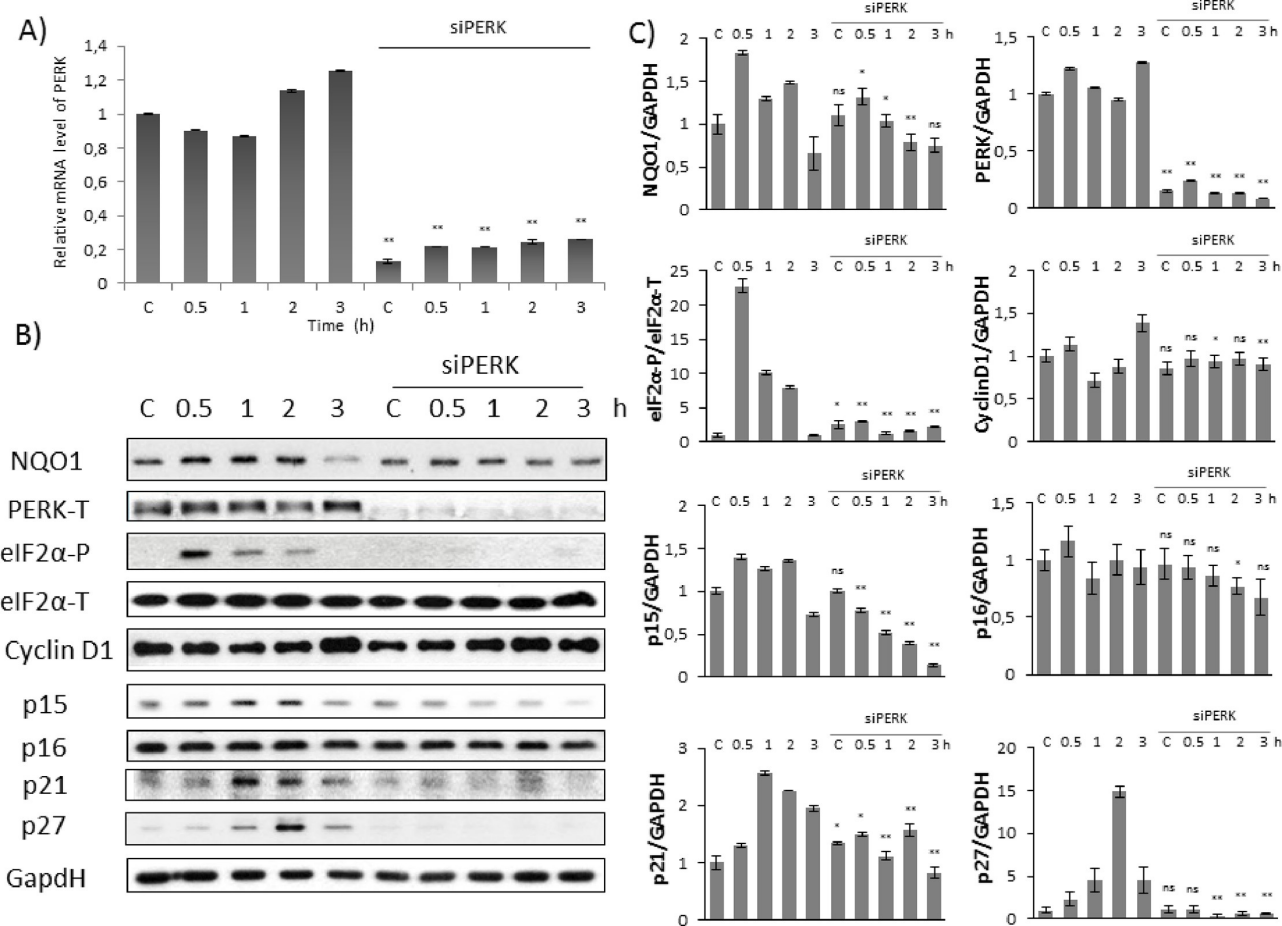
Silencing of PERK down-regulates Cyclin D1 inhibitors at early stage of oxidative stress. Time dependency of oxidative stress treatment was followed with/without a background of PERK silencing. HEK293T cells were cultured with TBHP for 0.5-1-2-3 hours, while *PERK* gene expression was depleted by *PERK* siRNA. **(A)** Testing the effectiveness of RNA silencing. PERK mRNA level was followed by real-time PCR in cell culture containing *PERK* siRNA. GAPDH was used as a housekeeping gene. The intensity of PERK is normalised for GAPDH. **(B)** During oxidative stress the markers of NRF2 (NQO1), PERK (PERK-T, eiF2α -P), Cyclin D1 and its inhibitors (p15, p16, p21, p27) were followed by immunoblotting. GAPDH was used as a loading control. **(C)** Densitometry data represent the intensity of NQO1, PERK-T, Cyclin D1, p15, p16, p21 and p27 normalised for GAPDH and eiF2α-P normalized for total level of eiF2α. For each of the experiments, three independent measurements were carried out. Error bars represent standard deviation; asterisks indicate statistically significant difference from the control: * p < 0.05; ** p < 0.01.

In order to explore further the oxidative stress response mechanism first we studied the effect of mild oxidative stress (100 μM TBHP) on HEK293T cells in time-dependent manner combined with/without silencing of PERK with siRNA. To confirm that PERK silencing was effective, the expression level of its mRNA was followed by real-time PCR (Fig 3A). Cells growing 0.5, 1 and 2 hours in 100 μM TBHP have shown a significant eiF2α phosphorylation suggesting that PERK pathway got activated; however eiF2α became dephosphorylated upon 3 hours long oxidative stress. No eiF2α phosphorylation was observed when PERK was silenced with siRNA in HEK293T cells.

Next, we followed the Cyclin D1 inhibitors (i.e. p15, p16, p21 and p27) by immunoblotting. As shown in Fig 3B and 3C, the level of p15, p21 and 27 resulted in a time-dependent increase, suggesting their essential role with respect to oxidative stress. p15, p21 and p27 performed a fast and significant activation peak already after 1 hour TBHP treatment, meanwhile Cyclin D1 level did not change at all. These results assume that Cyclin D1 might be regulated by their stoichiometric inhibitors at mild level of TBHP and the induction of cell cycle block might be achieved by this posttranslational modification at early stage of oxidative stress. In PERK silenced cells no p15, p21 and p27 activity was detected further confirming that PERK has a crucial role in regulating Cyclin D1 via its inhibitors upon oxidative stress. Interestingly, p16 level did not show any increase during TBHP treatment (Fig 3B and 3C). Besides that, PERK silencing did not have any effect on p16 protein level, suggesting that p16 was not part of the oxidative stress response mechanism.

Collectively, these results suggest that PERK has an essential role in controlling cell cycle progression via inducing Cyclin D1 inhibitors at initial stage of permanent mild oxidative stress.

### NRF2 is essential to up-regulate Cyclin D1 inhibitors upon low level of oxidative stress

**Fig 4:**
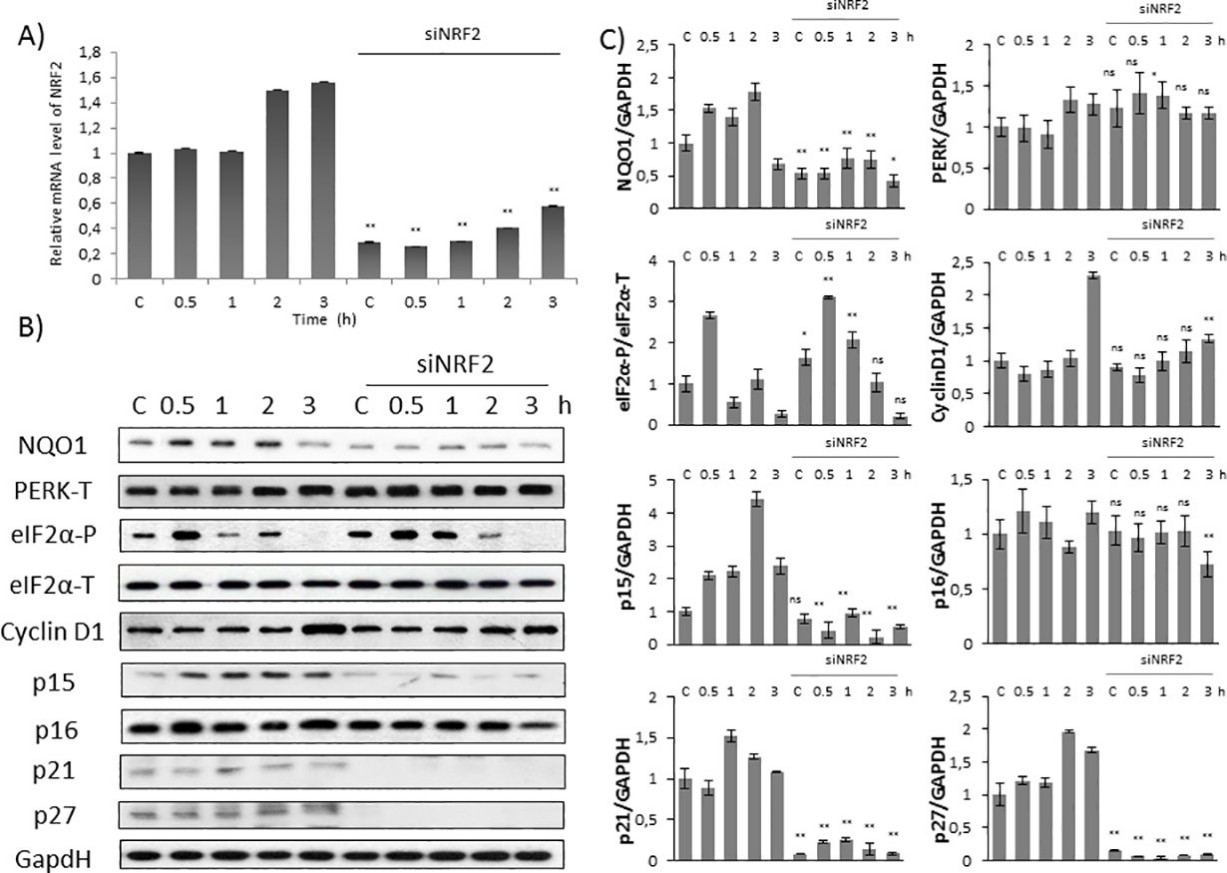
Silencing of NRF2 down-regulates Cyclin D1 inhibitors at early stage of oxidative stress. Time dependency of oxidative stress treatment was followed with/without a background of NRF2 silencing. HEK293T cells were cultured with TBHP for 0.5-1-2-3 hours, while *NRF2* gene expression was depleted by *NRF2* siRNA. **(A)** Testing the effectiveness of RNA silencing. NRF2 mRNA level was followed by real-time PCR in cell culture containing *NRF2* siRNA. GAPDH was used as a housekeeping gene. The intensity of NRF2 is normalised for GAPDH. **(B)** During oxidative stress the markers of NRF2 (NQO1), PERK (PERK-T, eiF2α -P), Cyclin D1 and its inhibitors (p15, p16, p21, p27) were followed by immunoblotting. GAPDH was used as a loading control. **(C)** Densitometry data represent the intensity of NQO1, PERK-T, Cyclin D1, p15, p16, p21 and p27 normalised for GAPDH and eiF2α-P normalized for total level of eiF2α. For each of the experiments, three independent measurements were carried out. Error bars represent standard deviation; asterisks indicate statistically significant difference from the control: * p < 0.05; ** p < 0.01.

Next, our goal was to verify the role of NRF2 in cell cycle progression upon oxidative stress. The longer cells were treated with 100 μM TBHP, the more NRF2 mRNA was present in HEK293T cells (Fig 4A). Decrease in NRF2 mRNA level was not observed when TBHP treatment was combined with PERK siRNA (Fig 2B); however its well-known target, NQO1 did not show a significant increase on protein level even after 3 hours TBHP treatment (Fig 3B and 3C). These results suggest that NRF2 cannot become catalytically active in the absence of PERK assuming that NRF2 might require some PERK-dependent modification upon oxidative stress.

Since NRF2 activity seems to be constantly low in the absence of PERK, a question immediately arises, namely, what can be the effect of PERK on NRF2. Various online available databases (such as MINT, IntAct) indicate an observable interaction between PERK and NRF2 suggesting a PERK-dependent regulation on NRF2 [34]. Cullinan et al. have also recently identified NRF2 as a PERK substrate [24, 25]. They claimed that ER stress-dependent PERK induction is essential both for dissociation of cytoplasmic NRF2/KEAP1 complex and subsequent NRF2 nuclear import [24]. Cullinan et al. have suggested that PERK might control NRF2 activity by phosphorylation [24], and here we also assume that NRF2 gets enhanced in a PERK-dependent manner (Fig 3B and 3C).

Besides the down-regulation of Cyclin D1 inhibitors (i.e. p15, p21, p27), NRF2 activity was also suppressed in the absence of PERK in response to oxidative stress. However, it was not clear whether NRF2 is able to regulate the stoichiometric inhibitors of Cyclin D1 to induce cell cycle arrest upon oxidative stress. Recently, by a global mapping of binding sites for NRF2 Malhotra et al. have shown that NRF2 can bind to the promoter of p15, p21 and p27 [35]. They also claimed that NRF2 might have a positive effect on these stoichiometric inhibitors of Cyclin D1, although further studies were also suggested. Therefore we investigated the direct role of NRF2 on Cyclin D1 inhibitors (p15, p16, p21 and p27) upon oxidative stress.

In order to further evaluate the importance of NRF2 in cell cycle progression, we analysed the time-dependency of addition of 100 μM TBHP in HEK293T cells pre-treated with NRF2 siRNA (Fig 4). To confirm that the silencing was successful NRF2 mRNA level was tested by real-time PCR (Fig 4A). In addition, by immunoblotting the absence of NQO1 protein level in TBHP treatment combined with NRF2 siRNA was also verified that NRF2 did not have any activity in this case (Fig 4B and 4C). As shown in Fig 4B and 4C, eiF2α phosphorylation was observed in a time-dependent manner during TBHP treatment suggesting that NRF2 does not have any feedback effect on neither PERK nor its target.

To verify the relationship between NRF2 and Cyclin D1 inhibitors upon prolonged but mild oxidative stress, the expression level of Cyclin D1, p15, p16, p21 and p27 was analysed by Western blot in HEK293T cells pre-treated with/without siNRF2 (Fig 4B and 4C). Both Cyclin D1 and p16 level were constant during TBHP treatment even in the absence of NRF2 suggesting that NRF2 did not affect these proteins at early stage of mild oxidative stress. While the protein level of p15, p21 and p27 has significantly increased upon prolonged oxidative stress in a time-dependent manner, we observed the complete diminish of these stoichiometric inhibitors in NRF2-silenced cells (Fig 4B and 4C).

These results imply that NRF2 is required for initiation of early stage of cell cycle block to induce Cyclin D1 inhibitors upon mild oxidative stress.

### Induction of Cyclin D1 inhibitors is always observed at early stage of oxidative stress

Finally, we attempted to determine whether the activation profile of Cyclin D1 inhibitors shows any differences at early stage of mild and excessive oxidative stress levels. Therefore HEK293T cells were treated with 100 or 300 μM TBHP and expression level of Cyclin D1 and its inhibitors (p15, p16, p21 and p27) were followed by immunoblotting.

**Fig 5:**
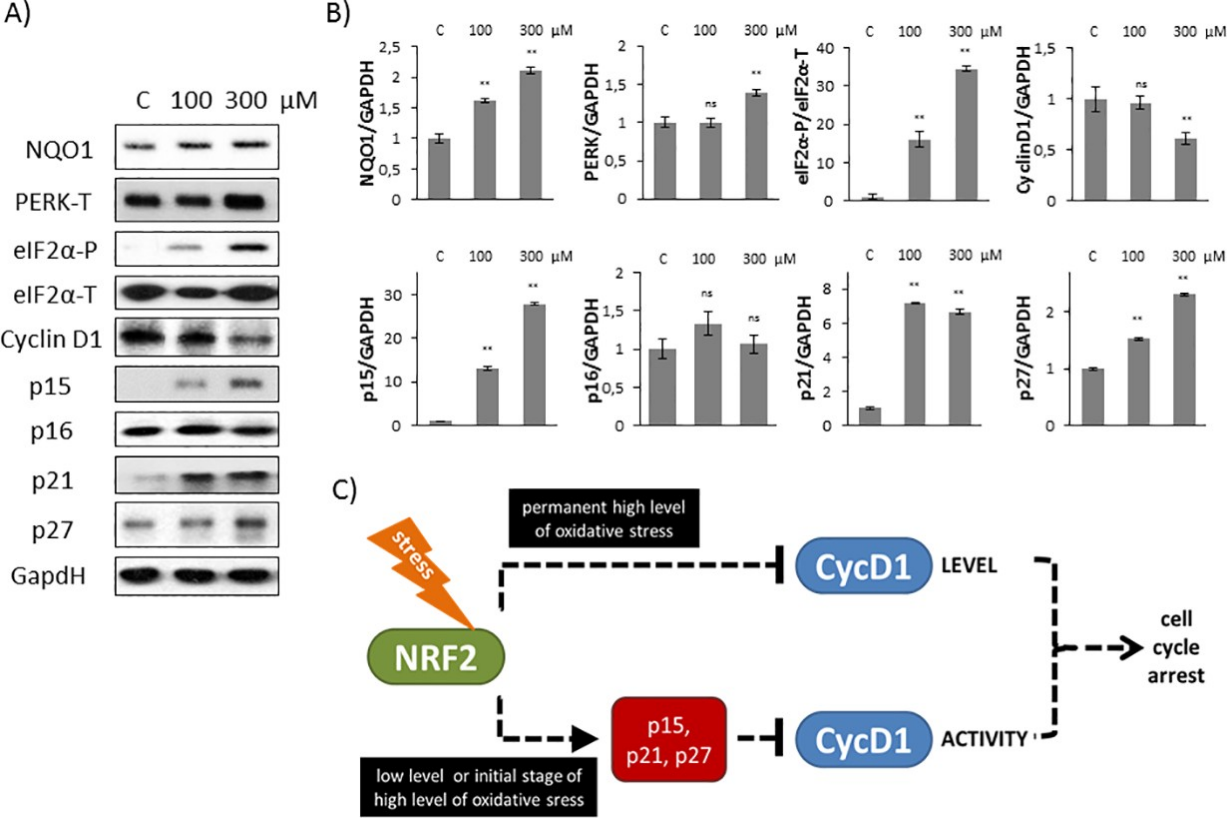
Short treatment with high level of oxidative stress induces Cyclin D1 inhibitors. **(A)** HEK293T cells were treated with 100 or 300 μM TBHP for 1.5 hour and the markers of NRF2 (NQO1), PERK (PERK-T, eiF2α -P), Cyclin D1 and its inhibitors (p15, p16, p21, p27) were followed by immunoblotting. GAPDH was used as a loading control. **(B)** Densitometry data represent the intensity of NQO1, PERK-T, Cyclin D1, p15, p16, p21 and p27 normalised for GAPDH and eiF2α-P normalized for total level of eiF2α. For each of the experiments, three independent measurements were carried out. Error bars represent standard deviation; asterisks indicate statistically significant difference from the control: * p < 0.05; ** p < 0.01. **(C)** The hypothetic control network of cell cycle regulatory system with respect to oxidative stress.

As shown in Fig 5A and 5B Cyclin D1 level got significantly decreased when 300 μM TBHP was added to HEK293T cells suggesting that cell cycle progression is definitively regulated through Cyclin D1 level at excessive level of oxidative stress. In contrast, p15, p21 and p27 levels resulted in a drastic increase at both mild and excessive oxidative stress levels, meanwhile p16 level was not affected at all. These results further confirm that although p16 is a well-known inhibitor of Cyclin D1, it does not have any role in controlling the early stage of cellular stress-response mechanism with respect to oxidative stress.

Our results suggest that the posttranslational regulation of Cyclin D1 by its inhibitors always occurs at early stage of oxidative stress cell supposing that besides controlling Cyclin D1 level its down-regulation is also essential.

## Discussion

Oxidative stress generated by internal metabolism or environmental toxicant exposure affects many cellular functions, including various signalling pathways. The transcription factor NRF2 is a key element of the main signalling pathway responsible to cell defence against oxidative stress. NRF2 is an emerging regulator of cellular resistance to oxidative attacks by controlling the transcription of hundreds of mainly cytoprotective genes. The role of NRF2 in maintaining the cellular redox balance is deeply explored, although its effect in cell cycle progression upon oxidative stress is not well-studied yet. Recently Pyo et al. have revealed that PERK pathway, one of the three main branches of unfolded protein response (UPR) is important to block cells in G2 phase with respect to oxidative stress [12]. Since it has been also shown that NRF2 activity is regulated by PERK [24], in this report we explored whether this cell cycle regulation is NRF2-dependent or not with response to oxidative stress.

Using HEK293T cell line we successfully set up a protocol when the initiation stage of cell cycle arrest was detected during oxidative stress. With TBHP we could find proper treatment when cells seemed to be already committed to cell cycle arrest, but has not blocked yet in G2 phase (Fig 1). Pyo et al have already shown that Cyclin D1 quickly got decreased at oxidative stress resulting in a cell cycle block in G2 phase [12]. We observed similar depletion of Cyclin D1 when the cells were treated with high level of TBHP; however no Cyclin D1 degradation was detected upon mild level of oxidative stress (Fig 2). This observation was further confirmed by the fact that Cyclin D1 level was even higher after 3 hours long treatment with 100 μM TBHP (Figs 3 and 4). This suggests that Cyclin D1 might get accumulate at the early stage of mild oxidative stress; therefore the control system requires a completely different mechanism to induce cell cycle block. Interestingly, the level of stoichiometric inhibitors of Cyclin D1 (i.e. p15, p21 and p27) has quickly shown a significant increase (Figs 2–4). This is the first study we have shown that cell cycle arrest got achieved via posttranslational modification of Cyclin D1 rather protein degradation upon mild oxidative stress.

To figure out whether in this system the above mentioned observation is connected to NRF2, the effect of both PERK and NRF2 was checked by silencing either PERK or NRF2 with its corresponding siRNA. Our results revealed that the absence of either of these proteins has a dramatic effect on oxidative stress response mechanism suggesting the abolition of cell cycle block. Namely, stoichiometric inhibitors of Cyclin D1 (i.e. p15, p21 and p27) remained inactive even after 3 hours long treatment with low level of TBHP (Figs 2–4). Here we verify that both PERK and NRF2 are essential for proper cell cycle progression upon oxidative stress.

Recently it has shown that key substrate of PERK, called eiF2α-P is crucial to promote Cyclin D1 degradation and therefore arrests cell cycle at G2 phase with respect to oxidative stress [12]. Cyclin D1 depletion was clearly observed by generating a high level of oxidative stress with H_2_O_2_ addition [12]; however they did not check the effect of a much weaker oxidative exposure. In this study we reveal that PERK pathway by inducing its other downstream target NRF2 controls cell cycle progression both at permanent mild and short but excessive level of oxidative stress. Although both the initiation of cell cycle arrest and an increase of eiF2α-P level were observed when cells were treated with 100 μM TBHP, no Cyclin D1 degradation was detected (Figs 1 and 2). Cyclin D1 degradation becomes significant even at early stage of excessive level of oxidative stress (i.e. using 300 μM TBHP), however the activation of p15, p21 and p27 was also observed (Fig 5A and 5B) suggesting that the stoichiometric inhibitors always play an essential role in oxidative exposure.

Here we assume that PERK controls cell cycle progression via both substrates (i.e. NRF2, eiF2α-P). NRF2-dependent and eiF2α-P-dependent pathways are not independent from each other rather they are complementary. At early stage of mild oxidative stress the control system quickly enhances the initiation of cell cycle arrest via enhancing Cyclin D1 inhibitors in an NRF2-dependent manner. With silencing of NRF2 upon oxidative stress here we show that this cell cycle arrest is eiF2α-P-independent (Fig 4). However, excessive level of oxidative stress turns on eiF2α-P-dependent Cyclin D1 degradation. Since Cyclin D1 inhibitors got activated even at high level of oxidative stress (Fig 5A and 5B), we suggest that NRF2-dependent stress response mechanism always plays an essential role in early stage of oxidative stress.

In this study we suggest that Cyclin D1 degradation takes time, therefore the stress response mechanism requires a much faster answer via posttranslational modification. Namely, the control network up-regulates the stoichiometric inhibitors of Cyclin D1 to guarantee a rapid commitment to cell cycle arrest if the exposure of oxidative agent is intolerable. Here we also suppose that the inhibition of Cyclin D1 is followed later by the down-regulation of Cyclin D1 upon excessive level of oxidative stress (Fig 5C). Meanwhile Cyclin D degradation is not observed at mild oxidative stress even after 3 hours. Since the conditions are not so drastic, the system wants to be capable to re-activate Cyclin D1 and therefore it re-induces cell cycle progression as soon as the oxidative exposure gets diminished and the cells can successfully recover. Although NRF2 is a PERK substrate, here we show that mRNA level of PERK did not change upon oxidative stress in the absence of NRF2 (Fig 4). Besides, an intensive eiF2α phosphorylation was also observed with NRF2 siRNA suggesting that NRF2 does not have any effect on PERK activity either. Interestingly, Sandelin et al. have revealed that NRF2, as a transcription factor, can bind to the promoter region of PERK gene [34], supposing an NRF2-driven feedback effect on PERK. This result seems to be contradictory to our experimental data; however, we cannot rule out that this feedback effect of NRF2 on PERK is not manifested upon oxidative stress. This assumption needs further analysis in the future.

Besides p15, p16 is the main stoichiometric inhibitor of Cyclin D1 and plays a crucial role in controlling G1/S transition [36, 37]. Furthermore it was also confirmed that p15 is the mediator of TGF-β-induced cell cycle arrest and it might inhibit the growth of various tumour cells [38]. Here we show that, contrary to p15, p16 is not affected at all during early stage of either mild or excessive level of oxidative stress (Fig 2). In addition p16 seemed to be also constant when NRF2 was silenced by siRNA upon oxidative exposure (Fig 4B and 4C). Our results suggest that p16 is not taking part in cell cycle control during oxidative stress. It might be also conceivable that p16 has role in blocking cell cycle progression after a longer oxidative exposure.

To explore the role of NRF2 in controlling cell cycle progression upon oxidative stress is a pivotal point of our study, since the most commonly occurring complex diseases of the society (i.e. neurodegenerative diseases, metabolic diseases and carcinogenesis) generate oxidative stress in the human cellular system. E.g. neurons are blocked in G0 phase at physiological conditions, but cells quickly enter mitosis followed by apoptosis during Alzheimer’s disease [39, 40]. NRF2 level is low in the nucleus during Alzheimer’s disease, meanwhile strong oxidative stress is observed. We claim that our experimental condition, namely, NRF2 silencing combined with oxidative exposure, nicely mimics the Alzheimer’s disease on cellular level. Our data suggest that absence of Cyclin D1 inhibitors (i.e. p15, p21 and p27) might have an essential role in disturbing the cell cycle machinery during degradation of neurons. Therefore in this report we further suppose the cytoprotective effect of NRF2.
